# Neuroprotective effects of magnesium l-threonate in a hypoxic zebrafish model

**DOI:** 10.1186/s12868-020-00580-6

**Published:** 2020-06-26

**Authors:** Young-Sung Kim, Young Ju Won, Byung Gun Lim, Too Jae Min, Yeon-Hwa Kim, Il Ok Lee

**Affiliations:** 1 Department of Anesthesiology and Pain Medicine, Korea University Guro Hospital, Seoul, Korea; 2 Department of Anesthesiology and Pain Medicine, Korea University Ansan Hospital, Ansan, Korea; 3 Institute of Medical Science, Korea University Ansan Hospital, Korea University College of Medicine, Ansan, Korea

**Keywords:** Behavior, Glutamate, Hypoxia, Magnesium, Neuroprotection, Zebrafish

## Abstract

**Background:**

Hypoxia inhibits the uptake of glutamate (a major neurotransmitter in the brain closely related to cognitive function) into brain cells, and the initial response of cells to cortical hypoxia depends on glutamate. Previous studies have suggested that magnesium may have protective effects against hypoxic injuries. In particular, magnesium l-threonate (MgT) may increase magnesium ion concentrations in the brain better than MgSO_4_ and improve cognitive function.

**Methods:**

We evaluated cell viability under hypoxic conditions in the MgT- and MgSO_4_-treated human SH-SY5Y neurons, in vivo behavior using the T-maze test following hypoxia in MgT-treated zebrafish, activity of brain mitochondrial dehydrogenase by 2,3,5-triphenyltetrazolium chloride (TTC) staining, and protein expression of the excitatory amino acid transporter (EAAT) 4 glutamate transporter by western blotting.

**Results:**

Among the groups treated with hypoxia, cell viability significantly increased when pre-treated with 1 or 10 mM MgT (p = 0.009 and 0.026, respectively). Despite hypoxic insult, MgT-treated zebrafish showed preferences for the red compartment (p = 0.025 for distance and p = 0.007 for frequency of entries), suggesting memory preservation. TTC staining showed reduced cerebral infarction and preserved absorbance in the MgT-treated zebrafish brain after hypoxia (p = 0.010 compared to the hypoxia group). In addition, western blot showed upregulation of EAAT4 protein in the MgT treated group.

**Conclusions:**

Pre-treatment with MgT attenuated cell death and cerebral infarction due to hypoxia and protected cognitive function in zebrafish. In addition, MgT appeared to modulate expression of the glutamate transporter, EAAT4.

## Background

With a growing need to preserve cognitive function in an aging population, various drugs that can improve memory are being studied [1–3]. However, there is limited evidence of efficacy and limited indications for use of cognition-enhancing drugs including psychostimulants and glutamate activators. Misuse of such drugs may cause side effects and complications as well as social problems [3]. Therefore, it is necessary to establish more in-depth knowledge and scientific evidence to support the clinical applications of such drugs.

Hypoxic insults may result in rapid, irreversible, ischemic damage to neurons [4, 5], and a variety of functional deficits including cognitive impairments. Hypoxia is known to inhibit the migration of glutamate (one of the main neurotransmitters in the brain, closely related to cognitive function) into brain cells [1, 6, 7]. Previous studies confirmed that the initial response of cells to cortical hypoxia depends on glutamate [8]. Excitatory amino acid transporter 4 (EAAT4), a glutamate receptor, shows decreased immunoreactivity after hypoxic-ischemic damage [9].

Several studies have shown that magnesium sulfate (MgSO_4_) reduces levels of reactive oxygen species and inflammation following hypoxic injuries [10]. However, the increase in magnesium ion levels in the cerebrospinal fluid (CSF) following MgSO_4_ administration is limited by central nervous system regulation and the blood–brain barrier. Even in the case of a 150–200% increase in plasma magnesium concentrations in humans, that in the CSF increases by only 10–19% [11]. The relatively newly developed magnesium l-threonate (MgT) consists of a magnesium ion and threonate, which exists physiologically in the brain [12]. Unlike other magnesium compounds, MgT significantly increases magnesium ion levels in the CSF [13]. Previous studies have suggested several neuroprotective mechanisms of MgT in the rat using Alzheimer’s disease and neuropathy models [14–16]. Wei et al. showed that MgT prevented the reduction in glutamatergic synaptic transmission under Alzheimer’s disease-like pathological conditions [16]. We expected that their findings would be similarly applicable to the hypoxic model.

To assess the effects of magnesium on cognitive function, we used a hypoxic zebrafish model. Zebrafish demonstrate a 70% genetic similarity to human protein coding genes [17] and an 84% similarity in disease-related genes [18]; moreover, the zebrafish neurotransmitter system is similar to that of mammals [19]. This model has recently become regarded as an ideal vertebrate model that has a competitive edge in terms of time and cost for large-scale drug toxicity screening studies [20]. In our previous study, zebrafish were found to be useful in the evaluation of cognitive function including learning and memory [21].

In this study, we investigated whether MgT is associated with neuroprotection and improvement in cognitive function using a hypoxic zebrafish model. We hypothesized that magnesium would prevent hypoxia-induced cognitive dysfunction, decrease infarcted brain area, and upregulate the glutamate transporter, EAAT4. Further, we predicted that glutamate would have a role in the mechanism underlying MgT-induced neuroprotection.

## Methods

### Experimental animal

Adult zebrafish (4–6 months old, 2.5–3.5 cm long, 350 ± 50 mg of weight, wild type, Danio rerio) were used in the study. Animals were fed brine shrimp twice a day in a 28.5 °C tank, and were on a 14-h daytime and 10-h night cycle. The watercraft was equipped with a multi-stage filtration system with a sediment filter, post-carbon filter, fluorescent UV light, and sterilization filter (Zebrafish AutoSystem, Genomic Design, Daejeon, Korea). All procedures were reviewed and approved by the Committee on the Ethics of Animal Experiments of the Korea University Medical School (IACUC number KOREA-2018-0032). After behavior experiments, all zebrafish were anesthetized using MS-222 (tricaine, Sigma-Aldrich, St. Louis, Mo., USA) and euthanatized by decapitation.

### Drug administration

MgT (C_8_H_14_MgO_10_, molecular weight 294.50 g/mol, Doctor’s Best Inc., USA) and MgSO_4_ (anhydrous, molecular weight 120.37 g/mol, Biosesang Inc., Korea) were used in the study. MgT were centrifuged at 2000 revolutions per minute for 2 min in 30 mL of phosphate-buffered saline (PBS) to a concentration of 100 mM and a sample from the middle layer was diluted 10 times (10 mM) or 100 times (1 mM). MgSO_4_ was also centrifuged and diluted in PBS in the same way. Drug concentrations were determined based on physiological concentrations of magnesium [22, 23] and results of MgT toxicity experiments in zebrafish embryos described below.

### Evaluation of toxicity of MgT in zebrafish embryos

Zebrafish embryos were treated with 0 (control), 5, 25, 50, and 100 mM MgT (diluted in water) in a 6-well plate (ten embryos per well) and incubated at 26 °C. Two days later, heart rate and development were measured with a microscope. After 6 days, survival rate was evaluated.

### Hypoxic chamber

**Fig. 1:**
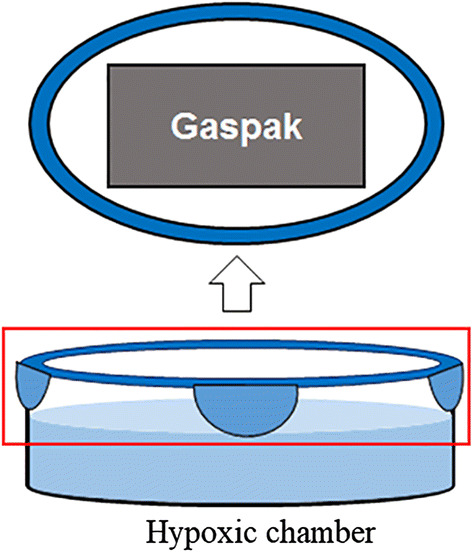
Schematic of the hypoxic chamber

The hypoxic chamber consisted of a closed glass box attached to an inner lid with a pack of Gaspak™ (Becton, Dickinson and Company, USA) (Fig. 1) filled with water such that the Gaspak™ was not immersed. The hypoxic chamber was closed the night prior to the experiment so as to reach at 1.0 ± 0.5 mg/L of dissolved oxygen (DO) as measured by a portable DO analyzer.

### Effects of MgT and MgSO_4_ on neurons

The human neuroblastoma cell line SH-SY5Y (ATCC^®^ CRL-2266™, American Type Culture Collection, USA) were used for in vitro experiments. After ensuring cells were healthy and free of contamination, and removing culture media, cells were treated with fetal bovine serum-free Dulbecco’s Modified Eagle Medium, and starved for two hours. Prewarmed 1 × trypsin solution was added to promote cell detachment in appropriate quantities (0.5 mL/10 cm^2^). When more than 90% of cells were detached, prewarmed complete growth media was added to inactivate the trypsin. After cell counting,  μL of cell suspension was added to a 96-well plate to obtain a final concentration of 7000 cells per well. The cells were treated with PBS (control), MgSO_4_ (1 or 10 mM) or MgT (1 or 10 mM) for 1 h. Media was changed, and the cells were incubated for two hours in a hypoxic chamber with no water (or a regular CO_2_ incubator as a control). To evaluate cell viability, 10 μL of cell counting kit-8 solution (CCK-8 solution, Dojindo Laboratories, Japan) was added to each well and absorbance was measured with a microplate reader (450 nm). To ensure reliability of the experimental results, experiments were repeated three times (each with 6 technical replicates per group).

### Hypoxic zebrafish model

Zebrafish exposed to hypoxia were evaluated using a 4-stage behavioral repertoire as previously described [24]. Briefly, fish were classified as stage 1 (swimming on the surface of the water), stage 2 (failure to attain normal posture), stage 3 (intermittent maintenance of opercular beats with brief movements), and stage 4 (dead). When a hypoxic zebrafish reached stage 3 it was immediately transferred to a normoxic chamber (DO: 7.0 ± 0.5 mg/L).

### Oral administration of PBS and MgT

Oral administration was performed by a professional after tricaine anesthesia [25]. Zebrafish were anesthetized in a water mixture containing 16.8 mg of tricaine per 100 mL, resuspended to minimize the anesthetic time, and administered 1 μL orally of PBS or MgT (10 mM) with a micropipette, taking care not to damage the mouth. This volume was chosen based on pilot data showing that zebrafish were likely to vomit solutions administered at volumes greater than 2 μL.

### Classification of the experimental group

**Fig. 2:**
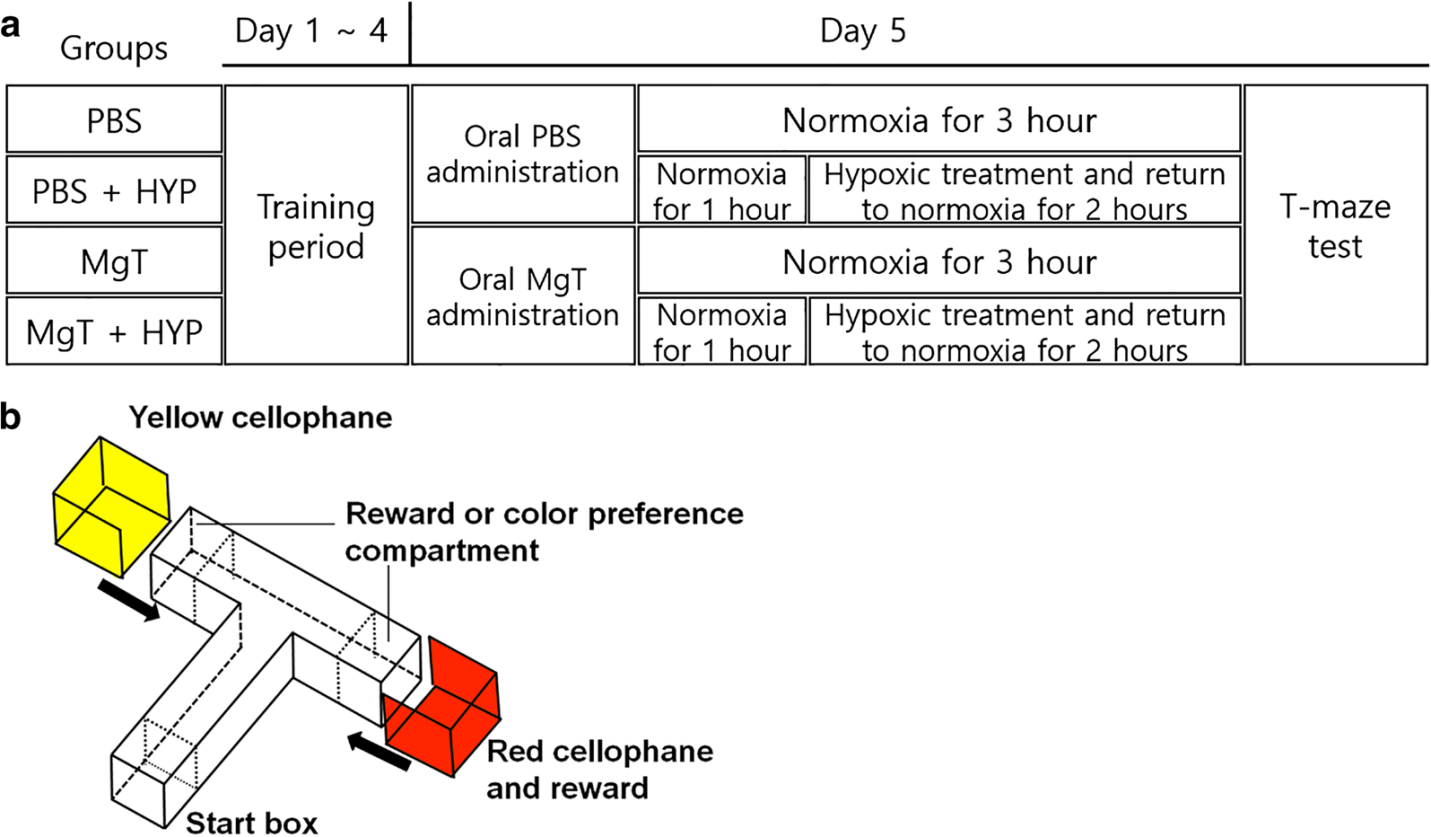
T-maze experiment. **a** Experimental groups and schedules. **b** Three-dimensional schematic of the T-maze. Colors indicate the two compartments: red for the target compartment and yellow for the opposite compartment

The experimental group was randomly divided into four groups: PBS = zebrafish that were orally administered 1 μL of PBS and remained in normoxia for 3 h; PBS + HYP = zebrafish that were orally administered 1 μL of PBS and placed in the hypoxic chamber for 1 h followed by normoxia for 2 h; MgT = zebrafish that were orally administered 10 mM of MgT and were in normoxia for 3 h; and the MgT + HYP group = zebrafish that were orally administered 10 mM of MgT and placed in the hypoxic chamber for 2 h followed by normoxia for 2 h (Fig. 2a). Randomization was performed using a web-based computer-generated list (www.randomization.com). The numbers were kept in opaque, sealed envelopes that were opened in the laboratory just before the experiment.

### Sample size calculation

Power analysis revealed that a minimum sample size of 10 for each group would be required to achieve a power of 80% at a significance level of 5%. Power analysis was calculated from results of our prior experiment [26]. To allow for exclusions, total sample size was prospectively set at 48 zebrafish (12 for each group).

### Color-added T-maze test

A T-shaped maze containing two arms and one stem was used to measure learning using color preference or compensation. A starting box (10 × 10 × 10 cm) was located at the bottom of the stem (50 × 10 × 10 cm) and two compartments (the “target” and “opposite” compartment; 10 × 10 × 10 cm each) were located at the ends of the maze arms (each 20 × 10 × 10 cm). Transparent sliding doors were used to separate the start box and the arms of the maze from the stem. During the training periods, sleeves made of red or yellow cellophane were fitted around the target and opposite compartments, respectively (Fig. 2b). To minimize bias, all the experiments started at 1–2 pm in a quiet dedicated place in the laboratory and all zebrafish were subjected to a habituation trial for 2 h prior to testing. Each zebrafish was trained once a day for four consecutive days. 20 µL of food (brine shrimp) was placed in the red cellophane compartment and each zebrafish was placed in the start box. On the fifth day, all zebrafish underwent memory testing, wherein all cellophane and food rewards were removed from the maze. All processes were recorded with a 4 K camcorder (Sony FDR-AX33, Sony Corporation, Japan) and analyzed using EthoVision XT software (Noldus Information Technology, Netherlands).

### Evaluation of MgT effects on zebrafish behavior

The evaluation indices for zebrafish behavior were as follows: time spent = total time spent in one of the compartments of the T-maze; distance moved = total horizontal distance moved in a compartment; and frequency of entries = the number of times the zebrafish entered one of the compartments.

Compartment preference was calculated using the following equation: preference = log(target compartment/opposite compartment). Therefore, 0 indicated no preference, and 1 indicated an index value in the target compartment 10 times higher than that of the opposite compartment. Conversely, a negative preference value indicated that the opposite compartment was preferred.

### 2,3,5-triphenyltetrazolium chloride (TTC) staining

TTC staining is a widely used method to measure hypoxic brain damage [27] and evaluate activity of brain mitochondrial dehydrogenase. To remove the brain, zebrafish were anesthetized using MS-222 (tricaine) and euthanatized by decapitation upon completion of behavior testing. For gross infarct size assessment, the brain was incubated in 1 mL of PBS containing 2% TTC (Sigma-Aldrich) for 40 min, followed by overnight incubation in 4% paraformaldehyde. The following day, the brain was imaged with a microscope. For absorbance measurement, the brain was incubated in TTC solution in a CO_2_ incubator for 100 min. The TTC solution was discarded and the brain was gently rinsed with 2–3 drops of dimethyl sulfoxide (DMSO)/ethanol (1:1 solution) and stored overnight in a 1.5 mL tube containing 1 mL of DMSO/ethanol solution. The following day, the absorbance of the DMSO/ethanol solution was measured with a spectrophotometer (Epoch, BioTek instruments, USA) and corrected by the brain weight.

### Western blot

To determine the mechanism of MgT in neuroprotection during hypoxia, we measured the expression of a glutamate transporter, excitatory amino acid transporter (EAAT) 4, by western blot. Following behavioral testing, zebrafish brains were homogenized in a lysis buffer (radio-immunoprecipitation buffer; Sigma) containing a protease inhibitor cocktail (Roche). Protein concentration was determined using the Bradford method. Proteins (20 μg) were separated by 8% sodium dodecyl sulfate polyacrylamide gel electrophoresis and transferred to membranes. The membranes were blocked with 5% skim milk in 1 × tris-buffered saline (TBS) at room temperature for 1 hour. The membranes were then incubated with a rabbit anti-EAAT4 antibody (ab41650, Abcam, Cambridge, MA) or rabbit anti-beta actin (A5441, Sigma-Aldrich) overnight at 4 °C. Membranes were washed three times in 1 × TBS + 0.05% Tween 20 and incubated with a 1:2000 (EAAT4) or 1:5000 (beta actin) dilution of horseradish peroxidase (HRP)-conjugated anti-rabbit immunoglobulin G (IgG) secondary antibody. An electro-chemiluminescence kit was used to develop the western blots (Amersham, Boston, MA, USA). Quantitative analysis of densitometry was performed using ImageJ (v. 1.52a, National Institutes of Health, USA).

### Statistical analysis

All data were analyzed using SPSS 22 (IBM, USA) and GraphPad Prism 6.0 (GraphPad, USA). Data are expressed as mean with standard error of the mean. Data were tested for normality using the Kolmogorov–Smirnov test. Parametric or non-parametric analysis was performed as appropriate. For MgT toxicity and survival data, a Kaplan–Meier survival curve was evaluated using the log-rank (Mantel-Cox) test. Cell viability was compared using one-way analyses of variance, and Tukey’s multiple comparison tests were used for post hoc analysis among the treated and untreated hypoxia groups. T-maze results were compared using a paired T test or Wilcoxon matched-pair signed rank test to determine differences in compartment preference. P-value < 0.05 was considered significant.

## Results

### Evaluation of toxicity of MgT in zebrafish embryos

**Fig. 3:**
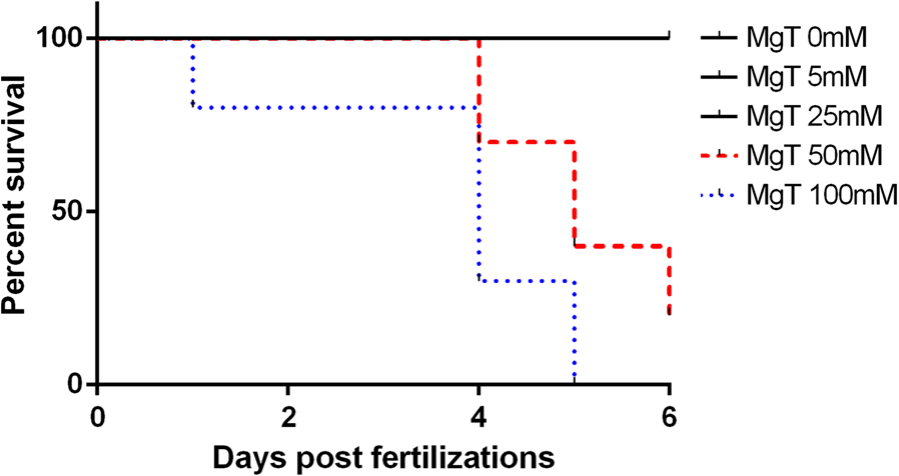
Kaplan Meier Survival Analysis. The log-rank (Mantel-Cox) test showed significant differences among the survival curves (p < 0.001)

Two embryos in the 100 mM group died on the day of MgT treatment. Two days after drug treatment, most zebrafish in the 100 mM group showed abnormal cardiac development. The remaining embryos that survived past the third day hatched. On the sixth day, all zebrafish in the control, MgT (5 mM), and MgT (25 mM) groups survived (10 zebrafish in each group, survival rate of 100%). Two survived in the MgT (50 mM) group (survival rate of 20%) and no zebrafish survived in MgT (100 mM) group (survival rate of 0%) Kaplan–Meier survival analysis showed significant differences between doses (p < 0.001) (Fig. 3).

### Effects of MgT and MgSO_4_ on neuron cells

**Fig. 4:**
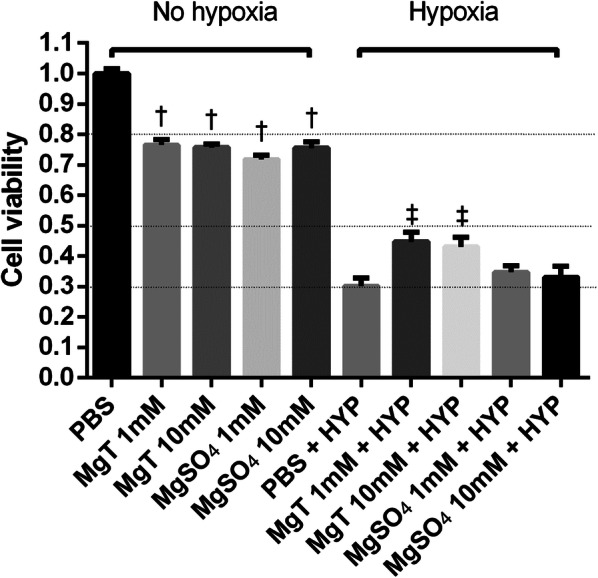
Effects of MgT and MgSO_4_ on neurons after normoxia (left) or hypoxia (right) in the presence or absence of MgT or MgSO_4_. MgT: magnesium l-threonate. The cells were treated with PBS (control), MgSO_4_ (1 or 10 mM) or MgT (1 or 10 mM) for 1 h. After the culture media were exchanged, the cells were incubated for 2 h in a CO_2_ incubator or a hypoxic chamber. The cellular experiments were repeated three times (each individual experiment contained 6 technical replicated, totaling 180 wells). Data are shown as mean ± SEM. ^†^p < 0.05 compared to the PBS group, ^‡^p < 0.05 compared to the PBS + HYP group. PBS: phosphate-buffered saline; MgT, magnesium l-threonate; HYP, hypoxia

In the groups that underwent hypoxia, cell viability was significantly decreased compared to the groups without hypoxic treatment. Among the normoxic groups, cell viabilities decreased when pretreated with MgT or MgSO_4_ (F (2, 51) = 84.65, p < 0.001 and F (2, 51) = 81.18, p < 0.001, respectively) (Fig. 4). On the contrary, among the hypoxic groups, cell viability significantly increased when pretreated with 1 mM or 10 mM MgT (F (2, 51) = 6.837, p = 0.0023) (Fig. 4). In the groups with 1 mM or 10 mM MgSO_4_, cell viability was not affected (F (2,51) = 0.6304, p = 0.5365). In both MgT and MgSO_4_, Concentration itself (1 or 10 mM) did not affect any significant difference, regardless of oxygen condition.

### Evaluation of MgT effects in Zebrafish behavior

Twelve zebrafish in the PBS group, 11 in the PBS + HYP group, 11 in the MgT group, and 11 in the MgT + HYP group were included in the behavior testing. In the hypoxia groups, the average time for the zebrafish to reach stage 3 of hypoxia was 10 ± 0.5 min.

The PBS group showed significantly longer time spent and distance moved in the target compartment than the opposite compartment (t (11) = 2.420, p = 0.034 and t (11) = 2.243, p = 0.046, respectively) while the frequency of entries was not significantly different (t (11) = 1.267, p = 0.231). There was no significant difference in time, distance, or frequency of entries between the target and opposite compartments in the PBS + HYP group (t (10) = 0.201, p = 0.845, t (10) = 0.011, p = 0.991, and t (10) = 0.686, p = 0.509, respectively) (Table 1). The MgT group also showed a significantly longer time and distance in the target compartment compared to the opposite compartment (t (10) = 2.901, p = 0.016 and t (10) = 2.507, 0.031, respectively) and the frequency of entries was unchanged (t (10) = 1.894, p = 0.088) (Table 1). The MgT + HYP group showed significantly higher distance and frequency of entries in the target compartment compared to the opposite compartment (t (10) = 2.641, p = 0.025 and (t (10) = 3.373, p = 0.007, respectively), and the difference in time was not significant (t (10) = 2.140, p = 0.058) (Table 1).

**Table 1:** T-Maze results

Group	Index	Target	Opposite
PBS (N = 12)	Time spent (sec)	93.2 ± 28.4*	65.1 ± 18.9
	Distance moved (cm)	566 ± 120*	426 ± 133
	Frequency (count)	17.9 ± 4.3	15.7 ± 6.6
PBS + HYP (N = 11)	Time spent (sec)	78.8 ± 39.7	75.0 ± 36.4
	Distance moved (cm)	466 ± 238	467 ± 270
	Frequency (count)	15.8 ± 7.0	14.0 ± 6.6
MgT (N = 11)	Time spent (sec)	115.2 ± 47.3*	57.7 ± 25.9
	Distance moved (cm)	513 ± 169*	351 ± 152
	Frequency (count)	14.3 ± 4.3	11.2 ± 3.8
MgT + HYP (N = 11)	Time spent (sec)	87.4 ± 49.3	46.7 ± 24.3
	Distance moved (cm)	483 ± 173*	304 ± 159
	Frequency (count)	17.9 ± 6.8*	12.7 ± 6.1

Values are mean ± SD. In the training period the target compartment was red + reward, and the opposite compartment was yellow. N represents the number of zebrafish. PBS, phosphate-buffered saline; MgT, magnesium l-threonate; HYP, hypoxia. *p < 0.05 compared to the opposite compartment

**Fig. 5:**
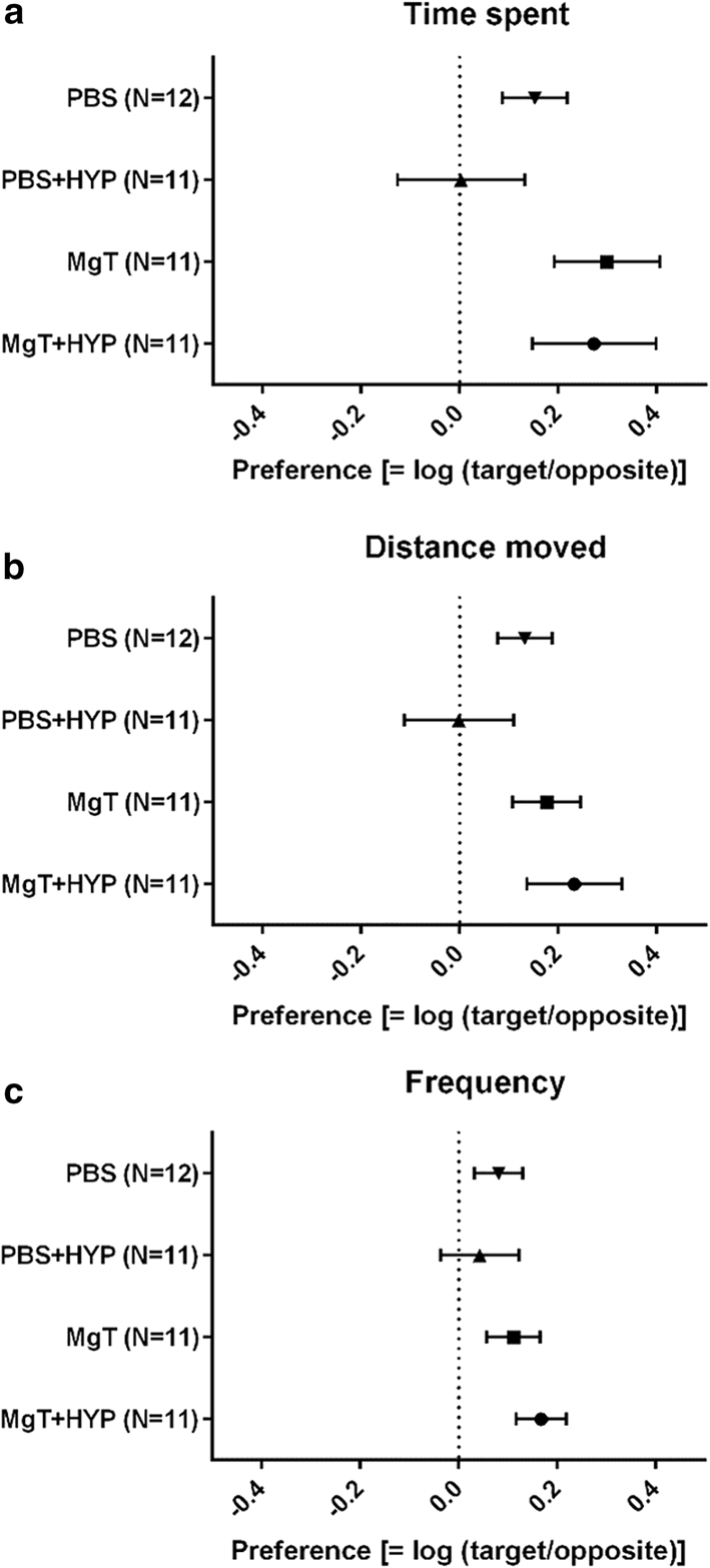
Preference for (**a**) time spent, (**b**) distance moved, and (**c**) frequency of entries into each compartment. Preferences were calculated as ‘Log (Target compartment/opposite compartment)’ from the zebrafish behavior test (see Table 1). Data are shown as mean ± SEM. The PBS + HYP group showed no preference in the behavior test. PBS, phosphate-buffered saline; MgT, magnesium l-threonate; HYP, hypoxia

Figure 5 shows the target compartment preference for time spent, distance moved, and frequency of entries. For all measures, the PBS + HYP group showed no compartment preference.

### TTC staining

**Fig. 6:**
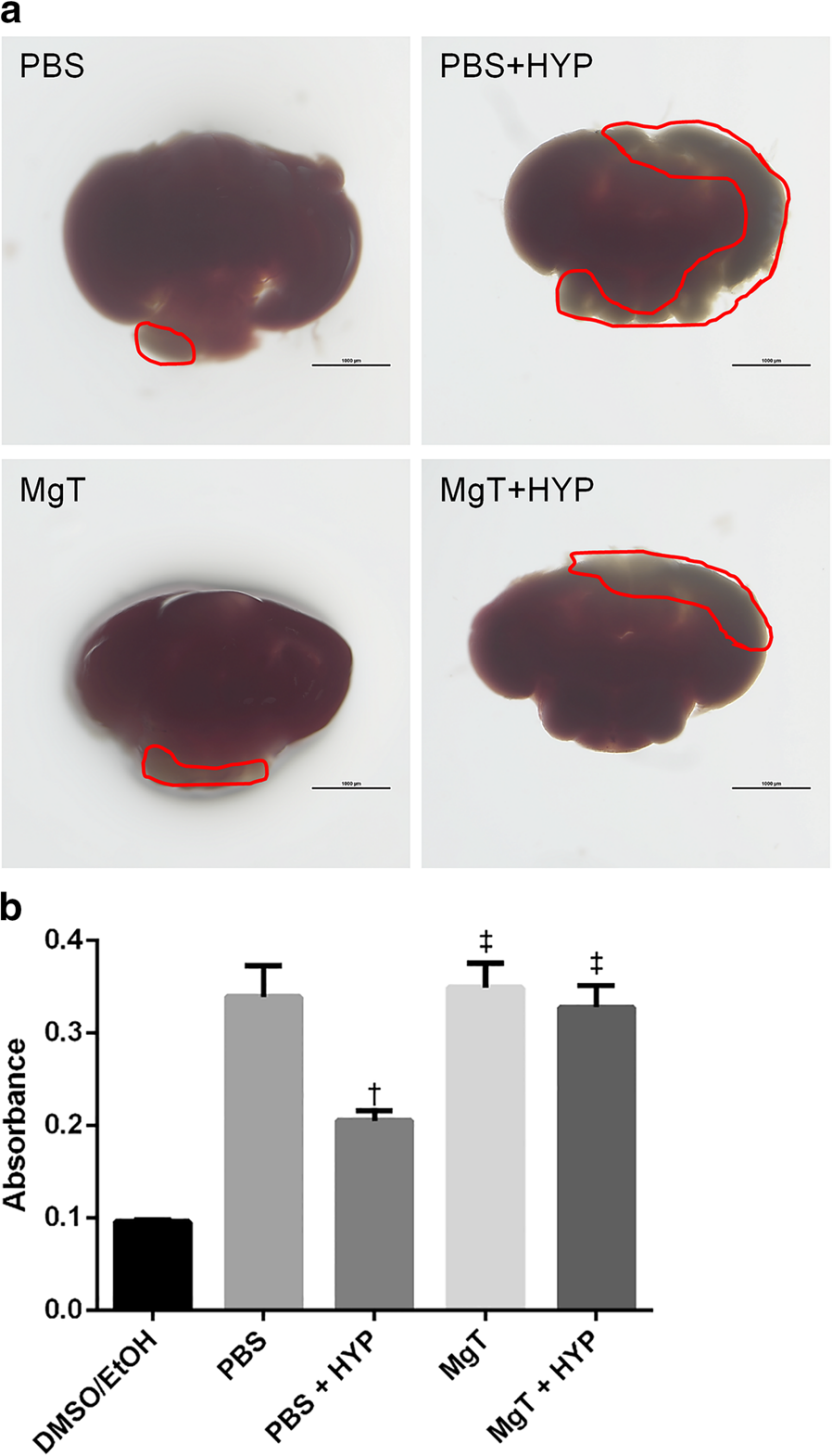
Zebrafish brain injury detected by TTC staining. **a** TTC-stained zebrafish brain sections: PBS, PBS + HYP, MgT, and MgT + HYP. Scale bar = 1000 um. The square indicates an unstained area. **b**Spectrophotometric measurement. Compared to the PBS group, the PBS + HYP group showed significantly low absorbance, while the MgT or MgT + HYP group did not. Eight brains in each group were analyzed. Data are shown as mean ± SEM. ^†^p < 0.05 compared to the PBS group, ^‡^p < 0.05 compared to the PBS + HYP group. PBS, phosphate-buffered saline; MgT, magnesium l-threonate; HYP, hypoxia. TTC, 2,3,5-triphenyltetrazolium chloride

Visualization with a microscope showed that the infarct area was reduced in the MgT + HYP group compared to the PBS + HYP group (Fig. 6a, Additional file 1). In addition, the PBS + HYP group showed significantly lower absorbance than the other groups (F (4,30) = 24.34, p < 0.001, adjusted p = 0.001 for PBS + HYP vs PBS, < 0.001 for PBS + HYP vs MgT and 0.002 for PBS + HYP vs MgT + HYP). There was no significant difference in absorbance between the PBS, MgT, and MgT + HYP groups (Fig. 6b).

### Western blot

**Fig. 7:**
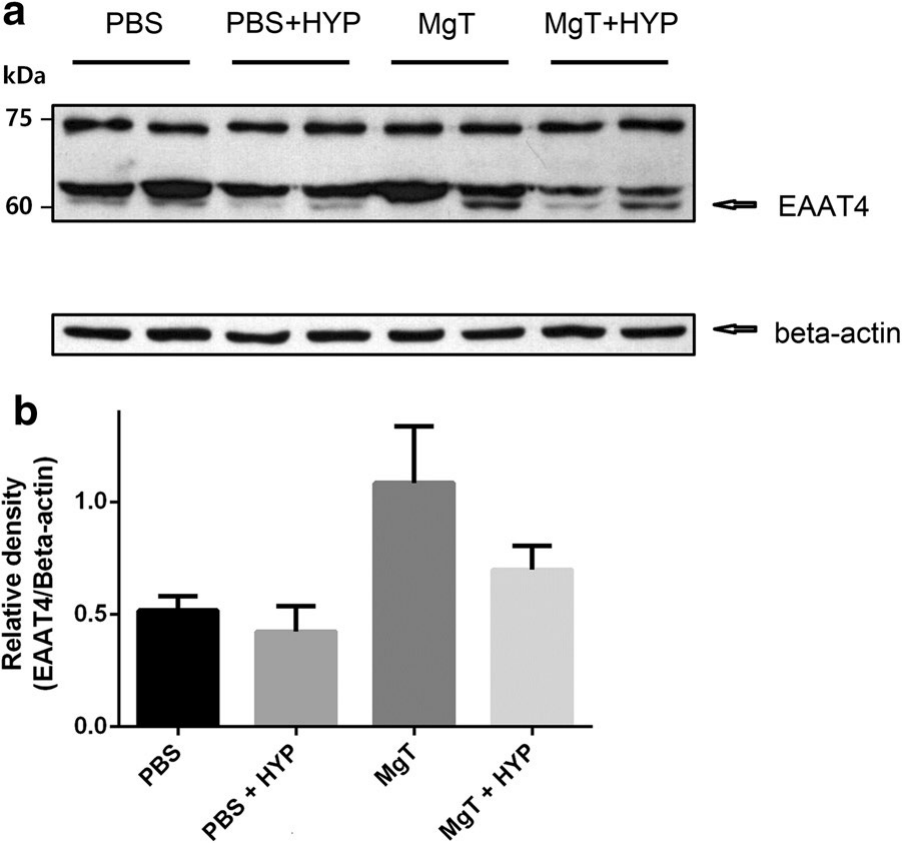
Upregulation of EAAT4. a Western blotting of EAAT4. b Optical density, normalized to beta actin. The predicted size of the EAAT4 protein was 61 kDa and that of beta actin was 42 kDa. Compared to the PBS group, an 18% decrease was observed in the PBS + HYP group, a 110% increase in the MgT group, and a 35% increase in the MgT + HYP group (representing a 65% increase compared to the PBS + HYP group and a 36% decrease compared to the MgT group). Data are shown as mean ± SEM. PBS, phosphate-buffered saline; MgT, magnesium l-threonate; HYP, hypoxia

Western blot revealed upregulation of EAAT4 in the MgT group. Compared to the PBS group, an 18% decrease was observed in the PBS + HYP group, a 110% increase was found in the MgT group, and a 35% increase was present in the MgT + HYP group (this was a 65% increase compared to the PBS + HYP group and a 36% decrease compared to the MgT group) (Fig. 7, Additional file 2).

## Discussion

In this study, we showed MgT may prevent hypoxia-induced cognitive dysfunction with decreased brain infarction and upregulated glutamate transporter (EAAT4). In addition to other studies which showed neuroprotective effects of MgT [12–16], our experiment suggested a new mechanism of MgT using a novel hypoxic zebrafish model.

Cerebral hypoxia–ischemia models have been widely used to evaluate mechanisms of neuroprotection [4]. The pathophysiology underlying hypoxic brain damage includes complex mechanisms including loss of ATP, excitotoxicity, production of free radicals, inflammation, over activation of the immune system, and cell death [28]. Although numerous mammalian studies have extensively investigated ischemic stroke, most have failed to develop therapeutic treatments for ischemia. Therefore, some progress may be made by studying hypoxic-tolerant organisms such as a fish [28].

The zebrafish is a relatively small and simple vertebrate organism, who’s genetic composition is similar to that of mammals, including humans. Thus, similar genes are likely to be associated with similar functions in humans [17]. Braga et al. reported spontaneous behavioral recovery in zebrafish after hypoxia [24]. In addition, several zebrafish models have been developed to demonstrate the usefulness of assessing cognitive function, learning, and memory [27, 29]. The zebrafish T-maze is based on visual discrimination learning [30]. Sison and Gerlai evaluated associative memory using visual perception in zebrafish [31]. Notably, zebrafish have specific color preferences [32]. We previously employed a similar zebrafish behavior model using color preferences in the absence of food rewards [21, 26]. Similar to our previous findings, a four-day training period was sufficient to allow zebrafish to develop a preference for a particular target compartment, even in the absence of colored cellophane and food during the testing period. Approximately 10 min of hypoxia reversed the effects of training and induced cerebral injury, consistent with our previous results [21, 26].

Magnesium is important for proper functioning in many tissues and organs including those of the cardiovascular, neuromuscular, and nervous systems. It plays an important role in synaptic plasticity [33] by reducing the calcium dependent post-burst after hyperpolarization of membrane potential, and regulating voltage-dependent blockade of N-methyl-D-aspartate (NMDA) glutamate receptors [23, 34]. Previous studies have shown that an increase in magnesium ion concentration in the extracellular fluid causes long-term enhancement of synaptic plasticity in hippocampal neurons [35]. Thus, increased magnesium in the brain may improve cognitive function.

Nonetheless, McKee et al. [11] reported limited neuroprotective effects of MgSO_4_ in patients with acute brain injury. Intravenous MgSO_4_ administration appeared to be hindered by the blood–brain barrier, leading to low levels of magnesium in the CSF, reflective of brain bioavailability [11, 36]. Compared with MgSO_4_, MgT has a different molecular structure that consists of a magnesium ion and threonate. Interestingly, Slutsky et al. [13] showed that sodium-L-threonate with/without magnesium chloride did not affect memory, while MgT enhanced memory recall. Sun et al. [12] reported several threonate effects, including increased mitochondrial function, glutamatergic synapse density, and neuronal intracellular magnesium ions in hippocampal neuronal cultures. They suggested that threonate may induce magnesium ion transport into hippocampal neurons.

Although reliable increases in magnesium levels are mostly safe [13], magnesium overdose may result in adverse events including lowered blood pressure, slowed heart rate, and cardiac arrhythmia or arrest. Because there was no prior study regarding MgT concentration, we first evaluated MgT toxicity in zebrafish embryos. After confirming that an MgT concentration below 25 mM showed no adverse effect on development or survival, we then performed a cellular experiment. In aerobic conditions, MgT and MgSO_4_ did not alter viability of human neuronal cell cultures. However, in hypoxic conditions, MgT treatment showed significantly improved cell viability, while MgSO_4_ treatment was not significant. In addition, the concentration (1 mM or 10 mM) of either MgT or MgSO_4_, did not affect outcome. This finding suggests that the regulation of the magnesium occurs at a cellular level at a relatively wide concentration range. We then performed an in vivo experiment with MgT. As expected, zebrafish pretreated with MgT maintained a preference in time, distance, and frequency of entries to the target compartment after hypoxic insult. The absorbance of zebrafish brain after TTC staining in the MgT + HYP groups was significantly higher than those in the PBS + HYP group. This indicates that MgT preconditioning reduced brain infarction and protected against hypoxic insult, in agreement with the behavioral results.

During hypoxia, many changes, including glutamate alteration, NMDA receptor stimulation, and neuronal degeneration occurred [37]. There are several explanations for neuroprotective effects of magnesium. Stevenson et al. [10] suggested that magnesium protects the central nervous system from hypoxic injuries through the prolyl hydroxylase or factor inhibiting hypoxia-inducible factor pathways. They focused on a specific genetic pathway (ephrinB2a with a hypoxia-inducible transcription factor 1 pathway) in neurodevelopment of zebrafish embryos. Others focused on tumor necrosis factor alpha to explain the neuroprotective effect of magnesium [14, 15]. Other plausible explanations include stabilization of the cell membrane, maintenance of ionic homeostasis by attenuating reductions in Na^+^K-ATPase activity, neuronal effects via reduction in NMDA receptor-mediated calcium entry into the cell, and vascular effects by improving cerebral blood flow [11, 37].

**Fig. 8:**
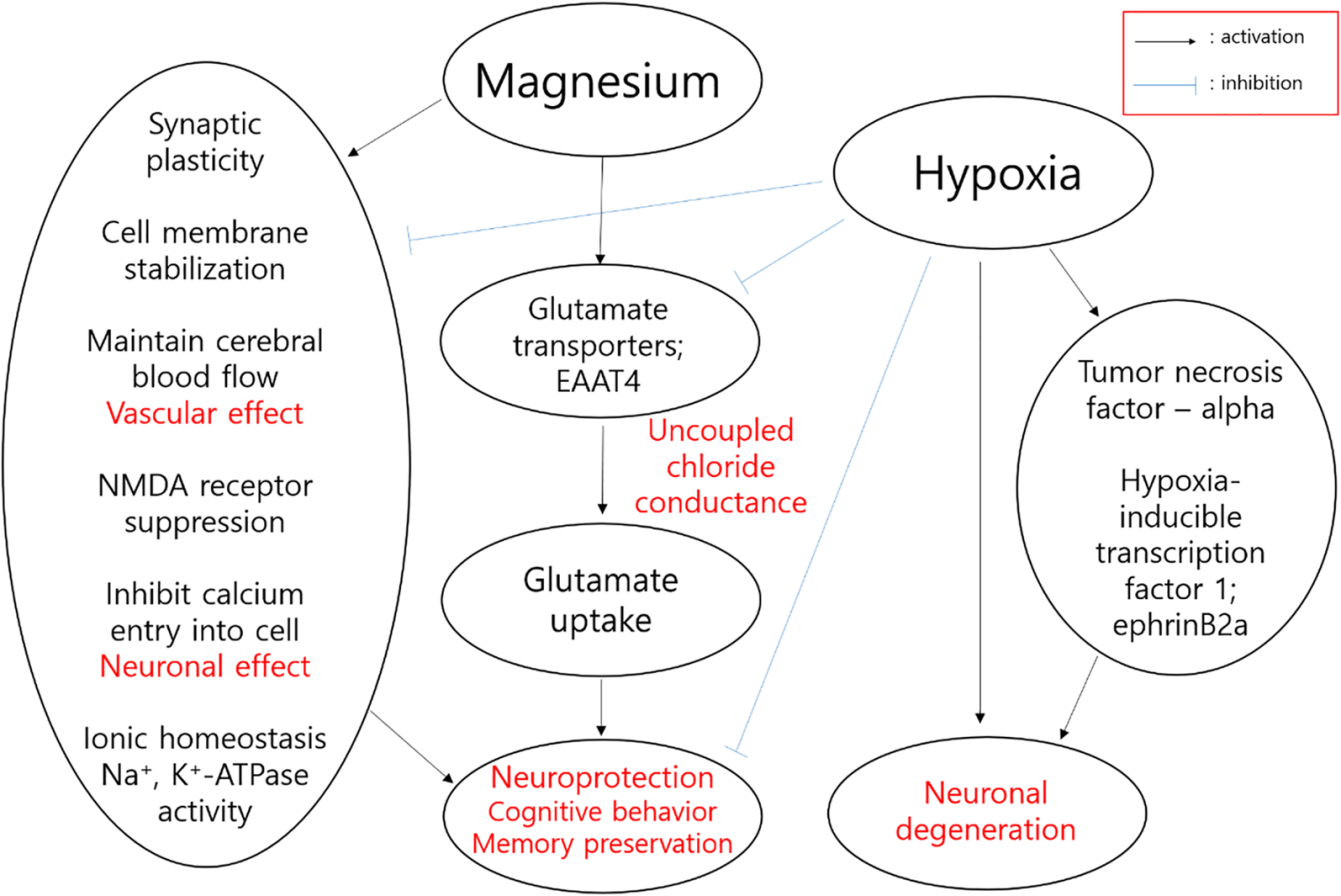
Schematic of the proposed mechanisms of hypoxia and magnesium. Black lines with arrows indicate activation while blue lines indicate inhibition. The red-colored texts represented the key concepts in the schematic diagram

In this study, we investigated the role of EAAT4 as a neuroprotective mechanism of magnesium. Once released into the synapse, glutamate is rapidly cleared by transporters (high-affinity EAATs) to limit excitotoxicity [38]. Keeping a low concentration of extracellular glutamate is also required for high signal-to-noise ratios during synaptic transmission [6]. Among the glutamate transporters, EAAT4 is expressed in several sites, including the cerebellum, hippocampus, and spinal cord [39–41]. Compared to EAAT subtypes 1-3, EAAT4 is associated with higher chloride conductance, which is not coupled to glutamate uptake, and therefore acts as an inhibitory glutamate receptor [39, 42]. By switching glutamate transport and chloride channel activity, EAAT4 may dampen cellular excitability during glutamate uptake and prevent a reduction in transport rate [42]. A previous study found that Purkinje cells die more easily in the event of EAAT4 deficiency after global brain ischemia [43]. Yi et al. [40] reported upregulated EAAT4 in rat hippocampal astrocytes 3–7 days after traumatic brain injury. In addition, Sachs et al. [44] suggested a role of EAAT4 in neuroprotection using a mutant neurodegenerative rat model. These findings suggest that EAAT4 may play a role, although the neuroprotective mechanisms are complex [45]. In this study, MgT groups showed upregulation of EAAT4 protein, which is approximately 61 kDa in size. Based on our results, MgT upregulates EAAT4 by 65–110%, whereas hypoxia downregulates EAAT4 by 18–36%. Taken together with previous experimental results, hypoxia may initially deplete EAAT4 and then induce EAAT4 reactivity in a later recovery phase [40]. Upregulation of EAAT4 by MgT appeared to protect the brain from hypoxic injury. A schematic diagram of the proposed mechanism of hypoxia and magnesium interaction is described in Fig. 8.

Western blotting demonstrated two unidentified protein bands expressed consistently in all groups. A consignment test also showed the two additional unknown bands mentioned above (data not shown). We referred to the antibody manufacturer’s guidelines to verify whether these bands may represent non-specific binding, or be due to incomplete antibody validation in the zebrafish. The antibody used was rabbit-derived, and validated in mice. One of the limitations of our study was that there was no suitable commercial antibody for zebrafish, making further evaluation of the mechanism limited. Moreover, it would be interesting to confirm the expression of EAAT4 several days following hypoxia. Another limitation of this study was that we performed tricaine anesthesia for oral administration of MgT or PBS prior to hypoxia and behavior testing. Although time under anesthesia was minimal, it may have influenced the outcomes via anesthetic preconditioning or toxic effects.

Despite these limitations, this study has several advantages. To the best of our knowledge, this study is the first application of drug-induced memory preservation in the hypoxic zebrafish behavior model. Because zebrafish are inexpensive and easy to manage, using our protocol may be useful to apply similar experiments designed to confirm the protective or toxic effects of other drugs. This finding suggests that hypoxic-tolerant organisms appear to have adaptive mechanisms to overcome hypoxic damage. Further experiments and observations are required to evaluate the specific mechanisms in order to contribute to the clinical application of potential treatments.

## Conclusion

This study showed that pretreatment with MgT, and subsequent upregulation of glutamate transporter EAAT4, has protective effects on neuronal survival, reduction in cerebral infarction, and preservation of learning and memory in zebrafish following hypoxia.

## Supplementary information

**Additional file 1: Figure S1.** Original version of TTC-stained zebrafish brain sections: PBS, PBS + HYP, MgT, and MgT + HYP. Scale bar= 1000 um (See Figure 6a).

**Additional file 2: Figure S2.** Original version of western blotting of EAAT4 (See Figure 7a).

## Data Availability

Data are available from Pf. Il-Ok Lee, Department of Anesthesiology and Pain Medicine, Korea University Guro Hospital, Seoul, Korea; E-mail: iloklee@korea.ac.kr for researchers who meet the criteria for access to confidential data.

## Abbreviations

CSF: Cerebrospinal fluid
DMSO: Dimethyl sulfoxide
DO: Dissolved oxygen
EAAT: Excitatory amino acid transporter
MgSO4: Magnesium sulfate
MgT: Magnesium l-threonate
PBS: Phosphate-buffered saline
TBS: Tris-buffered saline
TTC: 2,3,5-triphenyltetrazolium chloride
