# Supplementary figures and images for: Neuroprotective effects of magnesium l-threonate in a hypoxic zebrafish model
Source: BMC Neurosci. 2020 Jun 26;21:29. doi: 10.1186/s12868-020-00580-6 (PMC7318545; doi:10.1186/s12868-020-00580-6)

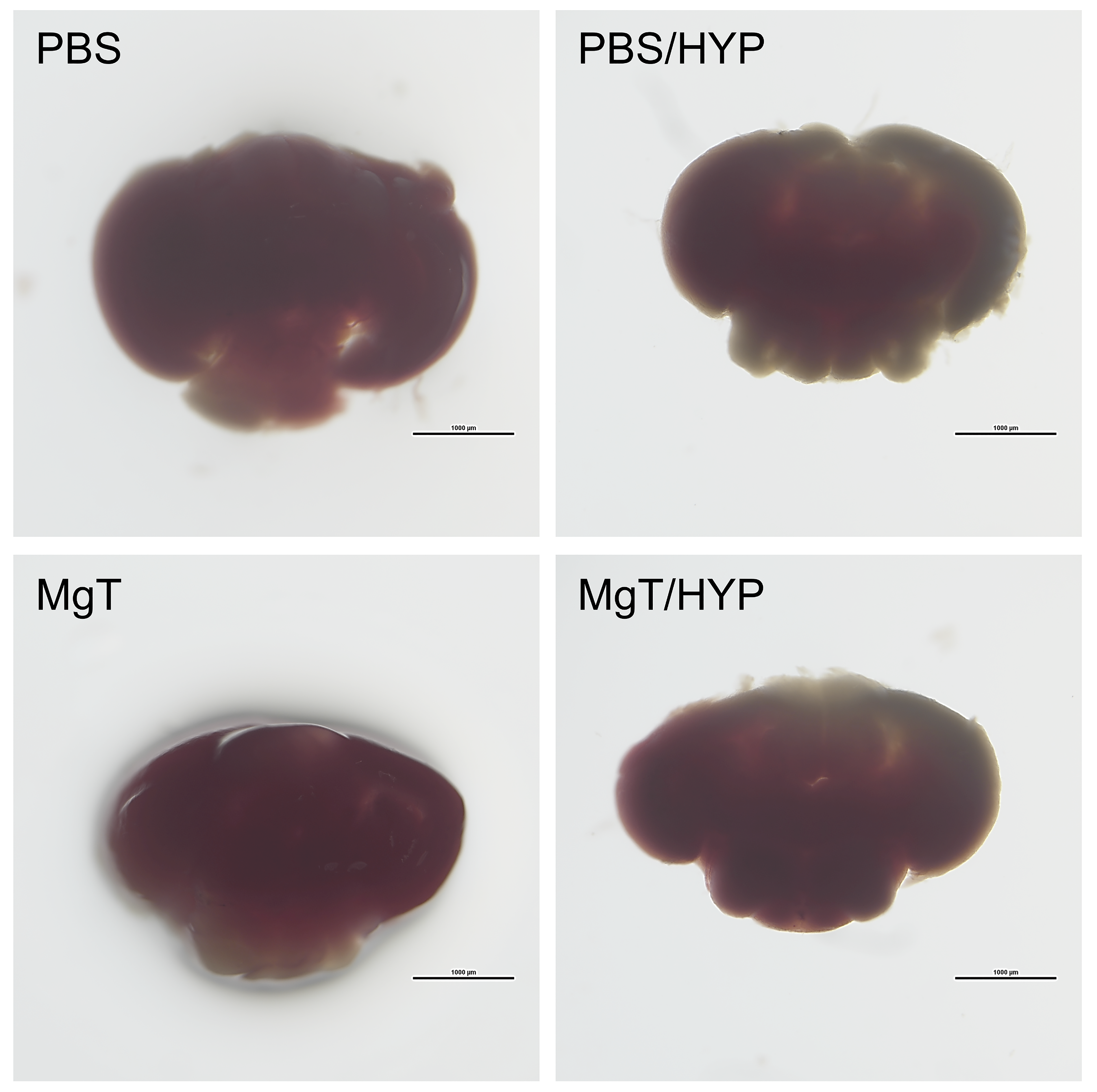

Supplement: Supplementary file 1 — Additional file 1: Figure S1. Original version of TTC-stained zebrafish brain sections: PBS, PBS + HYP, MgT, and MgT + HYP. Scale bar= 1000 um (See Figure 6a). [file 12868_2020_580_MOESM1_ESM.tif]

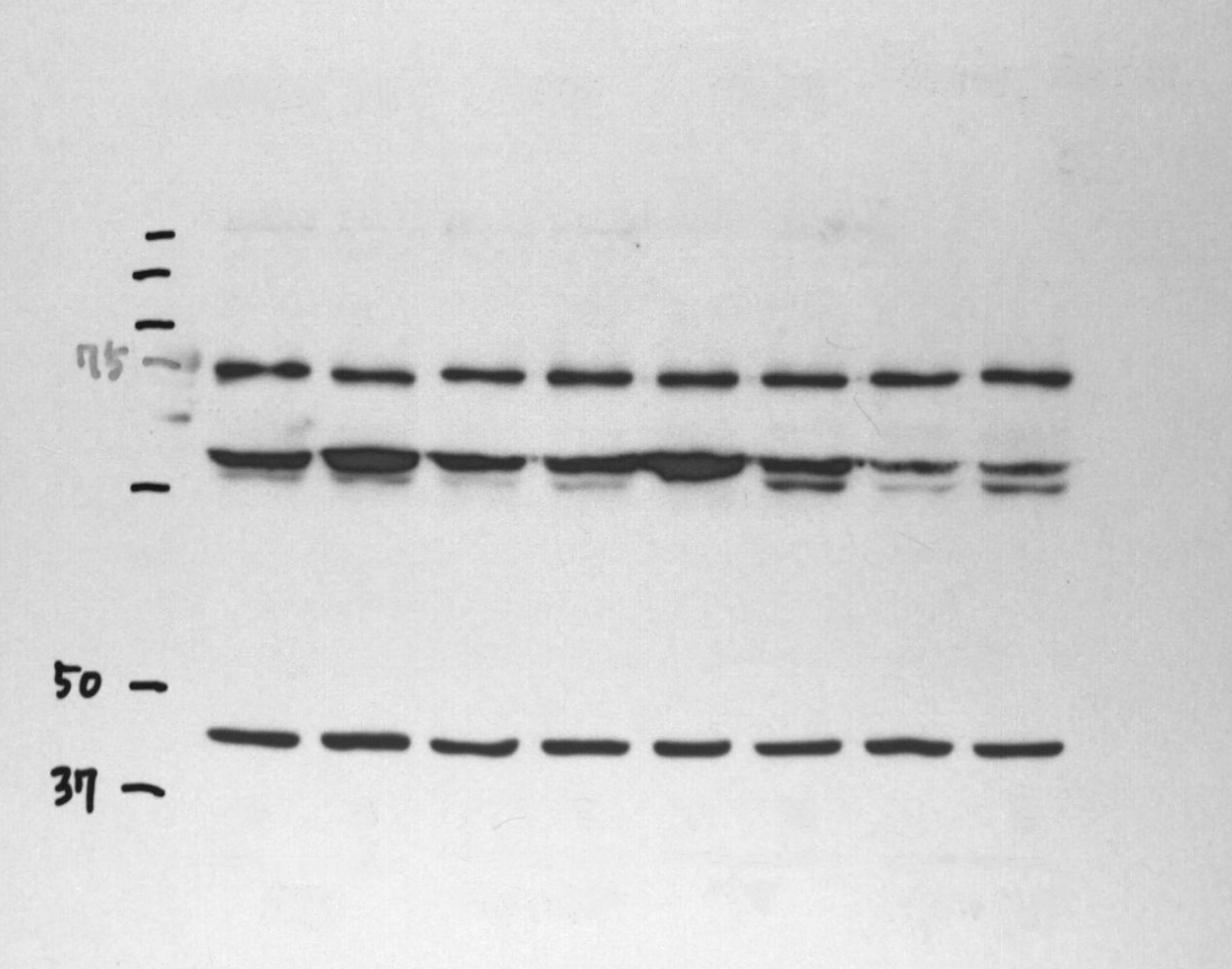

Supplement: Supplementary file 2 — Additional file 2: Figure S2. Original version of western blotting of EAAT4 (See Figure 7a). [file 12868_2020_580_MOESM2_ESM.tif]
